# Diminished circulating retinol and elevated α-TOH/retinol ratio predict an increased risk of cognitive decline in aging Chinese adults, especially in subjects with ApoE2 or ApoE4 genotype

**DOI:** 10.18632/aging.101694

**Published:** 2018-12-20

**Authors:** Xiaochen Huang, Huiqiang Zhang, Jie Zhen, Shengqi Dong, Yujie Guo, Nicholas Van Halm-Lutterodt, Linhong Yuan

**Affiliations:** 1 School of Public Health, Capital Medical University, Beijing 100069, P.R. China; 2 Department of Neurosurgery, Beijing Tiantan Hospital, Capital Medical University, Beijing 100050, China; 3 Department of Orthopaedics and Neurosurgery, University of Southern California, Keck Medical Center, Los Angeles, CA 90033, USA

**Keywords:** α-tocopherol, retinol, cognition, Apolipoprotein E, geriatrics

## Abstract

Objective: The current study evaluated the relationship between circulating fat soluble vitamin status and cognition in aging Chinese population.

Methods: A cross-sectional study was carried out in 1754 community residents aged 55-80 years aiming to evaluate the relationship between circulating α-tocopherol and retinol status and cognition. The effect of ApoE genetic polymorphism on the relationship between vitamins and cognition was also explored.

Results: Our results indicated that serum retinol status positively correlated with cognitive performance; while, serum α-tocopherol (α-TOH)/retinol ratio negatively correlated with cognitive performance. Mild cognitive impairment (MCI) subject demonstrated higher serum α-TOH status (*P* < 0.05), α-TOH/retinol ratio (*P* < 0.01) and lower retinol status (*P* < 0.01) than normal subjects. Subjects with ApoE4 genotype have lower serum retinol level (*P* < 0.05) and higher α-TOH/retinol ratio (*P* < 0.01) than subjects with ApoE3 genotype. MCI-ApoE4 carriers demonstrated the worst cognitive performance (*P* < 0.05) and exhibited higher serum TC, α-TOH and α-TOH/retinol ratio levels (*P* < 0.05), and lower LDL-C, retinol and lipid-adjusted retinol status (*P* < 0.05). MCI-ApoE2 subjects showed higher serum TC, HDL-C content and α-TOH/retinol ratio (*P* < 0.05); and lower serum retinol and lipid-adjusted retinol status (*P* < 0.05).

Conclusion: Lower circulating retinol and higher α-TOH/retinol ratio potentially predicts an increased risk for the development of cognitive decline in aging Chinese adults. ApoE2 or E4 carriers with higher circulating α-TOH/retinol ratio infer poor cognitive performance and an increased risk of developing MCI.

## INTRODUCTION

As powerful antioxidants, vitamin A (VA) and vitamin E (VE) play essential roles in maintaining normal brain function [1]. Growing number evidences indicate that greater dietary intake of VA and VE is associated with substantial reductions in AD risk; while, lesser intake of VA and VE may potentially contribute to neuro-degeneration with an increased risk of acquiring AD [2]. Animal-based experimental and population-based epidemiology studies have extensively highlighted the importance of maintaining optimal VA and VE nutritional status for normal cognitive function outcomes [3]. However, the supplementation of VE and VA provides limited clinical efficacy in the prevention and treatment of dementia [4]. Genetic heterogeneity has been reported as a determinant of in vivo vitamins status, which greatly contributes to the individual differences observed in response to vitamin supplementation [5]. Therefore, it has been speculated that an individual’s genetic background might determine individual’s sensitivity to the dietary supplementation of antioxidant vitamins.

Apolipoprotein E (ApoE) is a major regulator involved in lipid metabolism. ApoE is polymorphic, and the stability and susceptibility to degradation of ApoE has varied based on the ApoE genotype, leading to the unusual trend of increased serum lipids status observed in ApoE4 carriers [6]. The correlation of ApoE polymorphism and AD has been extensively reported [7]. The differences of serum lipid profile could only partially explain the different cognitive performance across ApoE genotypes [8]. However, there is still much to comprehend of how ApoE polymorphism interacts with other influencing factors (such as *in vivo* nutritional status) to affect cognition and even the development of dementia in aging population.

VA and VE share lipoproteins for their transportation, and their circulating status correlated with concurrent lipids [9]. As a result, the circulating concentrations of VA and VE might also be ApoE polymorphism dependent. Lower tissue α-tocopherol (α-TOH) concentration was found in ApoE4 mice compared with ApoE3 expressing mice [10]. A population-based study indicates that ApoE polymorphism is an independent determinant of plasma VA content [11]. Additionally, the presence of ApoE ε4 allele has been reported to play a prominent role in affecting serum VE concentration in cognitively healthy elderly individuals [12]. A study conducted in a non-Westernized population has depicted that the association between serum vitamin status and cognitive impairment could be potentially modulated by ApoE polymorphism [13]. These findings suggested the association of ApoE genetic variations, circulating vitamin status and cognition.

To date, the influence of ApoE genetic polymorphism regarding *in vivo* VA and VE status on cognition has not been fully investigated in aging Chinese population. Therefore, we carried out the present cross-sectional study with the main objective to analyze the association of circulating VA and VE status with cognitive performance. The modifying effect of ApoE genetic polymorphism on the relationship between antioxidant vitamins and cognition was also highlighted.

## RESULTS

### Demographic characteristics of the participants

Finally, total of 1754 individuals were included in the subsequent analysis. The mean age of the participants was 65.31 ± 6.30 years. The average BMI of the subjects was 25.34 ± 3.60 kg/m^2^. The average serum levels of α-TOH, γ-TOH and retinol were 27.3 ± 8.20 μmol/L, 4.30 ± 1.80 μmol/L and 1.92 ± 0.63 μmol/L respectively. Serum VA and VE levels were circulating lipids status related, therefore, the VA and VE levels were adjusted by lipid (total cholesterol + triglyceride, TC+TG) in the current study. And the average lipid-adjusted α-TOH, γ-TOH and retinol levels were 4.06 ± 0.98 μmol/mmol, 0.65 ± 0.24 μmol/mmol and 0.31 ± 0.10 μmol/mmol respectively (Table 1).

**Table 1:** Demographic characteristic of the participants.

**Demographic character**	***Total (n = 1754)***	**Demographic character**	***Total (n = 1754)***
**Age, mean ± SD**	65.31 ± 6.30	Smoking, n (Yes, %)	280 (16.0)
**Gender, n (%)**		Reading habit, n (Yes, %)	754 (43.0)
*Male*	568 (32.4)	AD family history, n (Yes, %)	152 (8.7)
*Female*	1186 (67.6)	**ApoE genotype, n (%)**	
**BMI (kg/m^2^), mean ± SD**	25.34 ± 3.6	*E2*	249 (14.2)
**Education, n (%)**		*E3*	1201 (68.5)
*Illiterate*	89 (5.1)	*E4*	304 (17.3)
*Primary school*	276 (15.7)	**Serum parameters, mean ± SD**	
*Junior high school*	768 (43.8)	*GLU (mmol/L)*	5.92 ± 1.86
*High school*	474 (27.0)	*TC (mmol/L)*	5.00 ± 1.03
*Junior college*	92 (5.2)	*TG (mmol/L)*	1.83 ± 1.41
*Undergraduate and above*	50 (2.9)	*LDL-C (mmol/L)*	2.88 ± 0.86
**Life style**		*HDL-C (mmol/L)*	1.43 ± 0.31
Physical activity, n (%)		*α-TOH (μmol/L)*	27.3 ± 8.20
*Never*	136 (7.8)	*γ-TOH (μmol/L)*	4.30 ± 1.80
*1-3 times/week*	210 (12.0)	*α-TOH /TC+TG (μmol/mmol)*	4.06 ± 0.98
*4-5 times/week*	198 (11.3)	*γ-TOH /TC+TG (μmol/mmol)*	0.65 ± 0.24
*everyday*	1210 (69.0)	*Retinol (μmol/L)*	1.92 ± 0.63
Alcohol drinking, n (Yes, %)	492 (28.1)	*Retinol/TC+TG (μg/mmol)*	0.31 ± 0.10

ApoE: Apolipoprotein E; AD: Alzheimer’s disease; SD: standard deviation; BMI: body mass index; GLU: glucose; TC: total cholesterol; TG: triglyceride; LDL-C: low density lipoprotein cholesterol; HDL-C: high density lipoprotein cholesterol; α-TOH: α-tocopherol; γ-TOH: γ-tocopherol.

### Serum parameters, ApoE genotype and food intake in normal and MCI subjects

According to the cut-off point of mild cognitive impairment (MCI) described in methods, 538 MCI subjects were screened. MCI subjects demonstrated higher serum glucose (GLU) (*P* < 0.05), total cholesterol (TC) (*P* < 0.05) and high-density lipoprotein cholesterol (HDL-C) (*P* < 0.01) and lower low-density lipoprotein cholesterol (LDL-C) (*P* < 0.01) levels than normal subjects. Higher serum α-TOH (*P* < 0.01) and lipid-adjusted α-TOH (α-TOH/TG+TC) (*P* < 0.05), and lower serum retinol (*P* < 0.01) and lipid-adjusted retinol (retinol/TG+TC) (*P* < 0.01) status were observed in MCI subjects. We did not detect the difference of ApoE genotype frequency between normal and MCI subjects (*P >* 0.05). MCI subjects also demonstrated higher serum α-TOH/retinol and γ-TOH/retinol ratio than normal subjects. Significant food intake difference was also observed between normal and MCI subjects, demonstrating by higher daily whole grains (*P* < 0.01), egg (*P* < 0.05) and lower vegetable (*P* < 0.01) intakes in MCI subjects (Table 2).

**Table 2:** Serum parameters, ApoE genotype and food intake in normal and MCI subjects.

**Parameters, ApoE genotype and food items**	**Normal (*n = 1171)***	**MCI (*n = 583)***	***P* value**
**Serum parameters**			
*GLU (mmol/L)*	5.85 (5.74, 5.96)	6.09 (5.94, 6.24)	0.014
*TC (mmol/L)*	4.96 (4.90, 5.02)	5.08 (5.00, 5.17)	0.014
*TG (mmol/L)*	1.83 (1.75, 1.92)	1.83 (1.71, 1.94)	0.934
*HDL-C (mmol/L)*	1.41 (1.39, 1.42)	1.48 (1.45, 1.50)	0.000
*LDL-C (mmol/L)*	2.91 (2.86, 2.96)	2.78 (2.71, 2.85)	0.003
*α-TOH (μmol/L)*	26.98 (26.51, 27.47)	28.09 (27.44, 28.77)	0.007
*γ-TOH(μmol/L)*	4.30 (4.20, 4.42)	4.42 (4.27, 4.58)	0.171
*α-TOH/TG+TC (μmol/mmol)*	4.06 (3.99, 4.11)	4.16 (4.09, 4.25)	0.020
*γ-TOH/TG+TC (μmol/mmol)*	0.65 (0.65, 0.67)	0.65 (0.65, 0.67)	0.430
*Retinol (μmol/L)*	1.99 (1.95, 2.02)	1.78 (1.71, 1.82)	0.000
*Retinol/TG+TC (mg/mmol)*	0.31 (0.31, 0.31)	0.28 (0.28, 0.28)	0.000
*α-TOH /retinol*	15.00 (14.61, 15.39)	17.50 (16.94, 18.0)	0.000
*γ-TOH /retinol*	2.36 (2.29, 2.44)	2.77 (2.66, 2.87)	0.000
**ApoE genotype, n (%)**			0.083
*E2*	150 (12.8)	99 (16.9)	
*E3*	813 (69.4)	388 (66.6)	
*E4*	208 (17.8)	96 (16.5)	
**Food items, (g/d)**			
*Fruit*	154.79 (148.52, 161.07)	154.61 (145.72, 163.51)	0.975
*Vegetable*	310.82 (303.05, 318.58)	287.55 (276.55, 298.56)	0.001
*Legume*	29.63 (28.07, 31.19)	30.51 (28.30, 32.73)	0.523
*Cooking oil*	29.52 (28.42, 30.62)	29.95 (28.40, 31.51)	0.656
*Fish*	19.96 (19.00, 20.91)	19.05 (17.70, 20.40)	0.283
*Whole grain*	33.83 (31.77, 35.88)	42.78 (39.86, 45.69)	0.000
*Red meat*	29.48 (27.77, 31.18)	30.77 (28.36, 33.19)	0.391
*Poultry*	13.92 (13.09, 14.75)	13.27 (12.09, 14.45)	0.377
*Nut*	17.16 (15.73, 18.59)	17.17 (15.15, 19.19)	0.994
*Milk*	128.55 (122.43, 134.67)	130.81 (122.13, 139.49)	0.676
*Egg*	31.23 (30.15, 32.31)	34.27 (32.74, 35.79)	0.002

The data were represented as mean (95% CI) or percentage. General Linear Model (GLM) was used for the comparison of serum parameters and food intakes. During the comparison of serum parameter, possible confounding factors including gender, age, BMI, smoking habit, physical activity, alcohol drinking, antioxidant supplement, diabetes and hyperlipidemia were adjusted. During comparison of daily food intakes, confounding factors including gender, age, BMI, smoking habit, physical activity and alcohol drinking were adjusted. Chi-square test was used for the comparison of ApoE genotype distribution among groups. MCI: mild cognitive impairment; TC: total cholesterol; TG: triglyceride; LDL-C: low density lipoprotein cholesterol; HDL-C: high density lipoprotein cholesterol; ApoE: Apolipoprotein E; α-TOH: α-tocopherol; γ-TOH: γ-tocopherol; MoCA: Montreal Cognitive Assessment. *P* < 0.05 was considered to be statistically significant.

### Correlation of serum vitamins and cognitive performance

Serum retinol status positively correlated with visual and executive (*r* = 0.188, *P* < 0.01), naming (*r* = 0.08, *P* < 0.01), attention (*r* = 0.073, *P* < 0.01), language (*r* = 0.187, *P* < 0.01), abstraction (*r* = 0.159, *P* < 0.01), memory and delayed recall (*r* = 0.161, *P* < 0.01) abilities, and global cognitive function (MoCA score) (*r* = 0.222, *P* < 0.01). Lipid-adjusted retinol status positively correlated with visual and executive (*r* = 0.168, *P* < 0.01), naming (*r* = 0.108, *P* < 0.01), attention (*r* = 0.084, *P* < 0.05), language (*r* = 0.154, *P* < 0.01), abstraction (*r* = 0.140, *P* < 0.01), memory and delayed recall (*r* = 0.137, *P* < 0.01) abilities and global cognitive function (MoCA score) (*r* = 0.206, *P* < 0.01). Serum α-TOH and γ-TOH status negatively correlated with visual and executive function (*r_α-TOH_* = -0.068, *P* < 0.01; *r_γ-TOH_* = -0.061, *P* < 0.05) and total MoCA score (*r_α-TOH_* = -0.055, *P* < 0.05; *r_γ-TOH_* = -0.058, *P* < 0.05). Serum α-TOH/retinol ratio negatively correlated with visual and executive (*r* = 0.168, *P* < 0.01), naming (*r* = 0.108, *P* < 0.01), attention (*r* = 0.084, *P* < 0.05), language (*r* = 0.154, *P* < 0.01), abstraction (*r* = 0.140, *P* < 0.01), memory and delayed recall (*r* = 0.137, *P* < 0.01) abilities, and global cognitive function (MoCA score) (*r* = 0.206, *P* < 0.01) (Table 3).

**Table 3:** Partial correlation coefficients between serum α-TOH and retinol status and cognition (n = 1754).

**Cognition**	**Retinol**	**Retinol /TG+TC**	**α-TOH**	**γ-TOH**	**α-TOH /TG+TC**	**γ-TOH /TG+TC**	**α-TOH /retinol**	**γ-TOH /retinol**
**Visual & executive**	0.188**	0.168**	-0.068**	-0.061*	-0.032	-0.039	-0.180**	-0.171**
**Naming**	0.080**	0.108**	-0.039	-0.096**	0.008	-0.069**	-0.093**	-0.137**
**Attention**	0.073**	0.084*	-0.031	-0.064**	0.020	-0.045	-0.058*	-0.092**
**Language**	0.187**	0.154**	-0.047	-0.015	-0.050*	-0.008	-0.160**	-0.131**
**Abstraction**	0.159**	0.140**	-0.013	0.002	-0.001	0.004	-0.115**	-0.092**
**Memory and delayed recall**	0.161**	0.137**	-0.015	-0.003	-0.020	-0.005	-0.111**	-0.103**
**Orientation**	-0.002	0.023	0.025	-0.057*	0.064	-0.043	0.038	-0.030
**MoCA Score**	0.222**	0.206**	-0.055*	-0.058*	-0.021	-0.039	-0.179**	-0.180**

Partial correlation analysis was used to explore the relationship between serum α-TOH, γ-TOH and retinol status with cognition. Factors including age, gender, BMI, smoking, alcohol and physical activity were adjusted during data analysis. MoCA: Montreal Cognitive Assessment; α-TOH: α-tocopherol; γ-TOH: γ-tocopherol; TG: triglyceride; TC: total cholesterol. *: *P* < 0.05; **: *P* < 0.01.

### Serum parameters, cognition and food intake according to lipid-adjusted retinol status

After grouping the subjects according to the quartile (Q1 - Q4) of lipid-adjusted retinol status, the difference of serum parameters, cognitive performance and food intakes between groups was compared. The highest serum TG and LDL-C status was observed in subjects with Q4 level of retinol status (*P* < 0.01). The highest serum HDL-C concentration was found in subjects with Q1 level of retinol status (*P* < 0.01). Following the increase of serum retinol status, cognitive performance demonstrated an increasing trend accordingly; and the best cognition was observed in the Q4 group. The dietary intake was different among the groups as well. The highest daily vegetable (*P* < 0.01) and the lowest fruit (*P* < 0.05), whole grains (*P* < 0.01), nuts (*P* < 0.01) and egg (*P* < 0.01) intake were observed in subjects with Q4 level of serum retinol status (Table 4).

**Table 4:** Serum parameters, cognition and food intakes according to lipid-adjusted retinol status (n = 1754).

**Parameters, cognition and Food intake**	**Retinol/TG+TC**				***P* value**
	**Q1 (n = 429)**	**Q2 (n = 455)**	**Q3 (n = 440)**	**Q4 (n = 430)**	
**Serum parameters (mmol/L)**					
*Glu*	6.26 (6.10, 6.42)	6.06 (5.91, 6.21)	5.78 (5.63, 5.93)^b^	5.60 (5.44, 5.76)^ab^	0.000
*TC*	5.55 (5.46, 5.64)	5.18 (5.10, 5.26)^a^	4.87 (4.78, 4.95)^ab^	4.40 (4.31, 4.49)^abc^	0.000
*TG*	2.53 (2.40, 2.66)	1.85 (1.73, 1.98^)a^	1.55 (1.43, 1.68)^ab^	1.39 (1.26, 1.52)^ab^	0.000
*HDL-C*	1.49 (1.46, 1.51)	1.46 (1.43, 1.49)	1.41 (1.38, 1.44)^ab^	1.36 (1.33, 1.39)^abc^	0.000
*LDL-C*	3.00 (2.92, 3.08)	2.87 (2.79, 2.95)^a^	2.90 (2.82, 2.98)^a^	2.72 (2.64, 2.80) ^abc^	0.000
**Cognition**					
*Visual-spatial and executive*	3.43 (3.32, 3.55)	3.66 (3.55, 3.77)^a^	3.75 (3.64, 3.86)^a^	3.93 (3.82, 4.05)^abc^	0.000
*Naming*	2.84 (2.80, 2.88)	2.86 (2.82, 2.90)	2.89 (2.85, 2.93)	2.94 (2.90, 2.98)^a^	0.009
*Attention*	5.31 (5.21, 5.41)	5.24 (5.15, 5.34)	5.33 (5.24, 5.44)	5.41 (5.31, 5.51)	0.148
*Language*	1.90 (1.81, 1.98)	1.97 (1.89, 2.05)	2.06 (1.98, 2.14)^a^	2.21 (2.13, 2.30)^abc^	0.000
*Abstraction*	1.45 (1.39, 1.52)	1.49 (1.43, 1.55)	1.54 (1.48, 1.61)	1.62 (1.55, 1.68)^abc^	0.007
*Memory and delayed recall*	2.59 (2.44, 2.73)	2.63 (2.49, 2.78)	2.73 (2.59, 2.87)	3.18 (3.03, 3.32)^abc^	0.000
*Orientation*	5.82 (5.76, 5.89)	5.77 (5.71, 5.84)	5.77 (5.70, 5.83)	5.86 (5.80, 5.93)	0.140
*MoCA score*	23.36 (22.95, 23.76)	23.66 (23.27, 24.04)	24.25 (23.86, 24.65)^ab^	25.51 (25.11, 25.91)^abc^	0.000
**Food Items, (g/d)**					
*Fruit*	162.47 (151.91, 173.04)	165.87 (155.75, 176.00)	153.16 (142.89, 163.44)	137.26 (126.68, 147.85)^abc^	0.010
*Vegetable*	287.63 (274.68, 300.57)	287.39 (274.98, 299.80)	300.96 (288.37, 313.55)	337.14 (324.17, 350.11)^abc^	0.000
*Legume*	30.71 (28.09, 33.34)	32.19 (29.67, 34.71)	29.43 (26.89, 31.98)	27.58 (24.94, 30.21)	0.090
*Cooking oil*	28.42 (26.57, 30.26)	29.05 (27.28, 30.82)	30.08 (28.28, 31.88)	31.14 (29.29, 32.99)	0.195
*Fish*	18.70 (17.09, 20.32)	20.47 (18.92, 22.01)	19.94 (18.37, 21.51)	19.33 (17.71, 20.95)	0.440
*Whole grain*	44.08 (40.67, 47.49)	41.86 (38.59, 45.12)	35.53 (32.21, 38.85)^ab^	25.37 (21.96, 28.79)^abc^	0.000
*Red meat*	32.04 (29.14, 34.93)	30.70 (27.93, 33.47)	29.57 (26.75, 32.38)	27.20 (24.30, 30.09)	0.132
*Poultry*	13.44 (12.03, 14.85)	14.28 (12.93, 15.63)	13.59 (12.22, 14.96)	13.52 (12.11, 14.93)	0.820
*Nuts*	22.64 (20.26, 25.01)	18.06 (15.78, 20.34)^a^	15.56 (13.25, 17.87)^a^	12.42 (10.04, 14.80)^a^	0.000
*Milk*	141.29 (130.97, 151.61)	124.35 (114.46, 134.23)^a^	126.89 (116.86, 136.93)	124.79 (114.46, 135.13)	0.072
*Egg*	35.50 (33.69, 37.30)	33.89 (32.16, 35.61)	31.14 (29.39, 32.90)^ab^	28.27 (26.47, 30.08)^abc^	0.000

The data were represented as mean (95% CI) or percentage. General Linear Model (GLM) was used for the comparison of serum parameters, cognitive performance and daily dietary intakes. During the comparison of serum parameter, possible confounding factors including gender, age, BMI, smoking habit, alcohol drinking, physical activity, diabetes and hyperlipidemia were adjusted; During the comparison of cognition, confounding factors including gender, age, BMI, smoking habit, physical activity, alcohol drinking, education level and AD family history were adjusted; During comparison of daily dietary intakes, confounding factors including gender, age, BMI, smoking habit, physical activity and alcohol drinking were adjusted. MoCA: Montreal Cognitive Assessment; Glu: glucose; TC: total cholesterol; TG: triglyceride; LDL-C: low density lipoprotein cholesterol; HDL-C: high density lipoprotein cholesterol; Q: quartile; a: comparing with Q1 group, *P* < 0.05; b: comparing with Q2 group, *P* < 0.05; c: comparing with Q3 group, *P* < 0.05.

### Serum parameters, cognition and food intake according to lipid-adjusted α-TOH status

Following an increasing trend in lipid-adjusted α-TOH status (from Q1 to Q4), the GLU and lipids concentration increased accordingly. Subjects in Q4 group showed higher serum GLU, TC, TG, HDL-C and LDL-C status (*P* < 0.01). Better memory and delayed recall ability (*P* < 0.05) and total MoCA score (*P* < 0.05) was found in subjects with Q2 level of α-TOH status (*P* < 0.05). Subjects in Q1 group demonstrated lower orientation ability (*P* < 0.05) compared to participants in Q3 and Q4 groups. For dietary intakes, subjects with Q4 level of serum α-TOH status have higher daily whole grains (*P* < 0.01) and milk intake (*P* < 0.05) (Table 5).

**Table 5:** Serum parameters, cognition and food intakes according to lipid-adjusted α-TOH status (n = 1754).

**Parameters, cognition and Food intake**	**α-TOH/TG+TC**									***P* value**					
	**Q1 (n = 431)**	**Q2 (n = 450)**		**Q3 (n = 426)**		**Q4 (n = 447)**				
**Serum parameters (mmol/L)**											
*GLU*	6.07 (5.91, 6.23)		5.93 (5.77, 6.08)		5.87 (5.71, 6.03)		5.85 (5.69, 6.00)		0.207				
*TC*	5.33 (5.23, 5.42)		5.08 (4.99, 5.16)^a^		4.97 (4.88, 5.06)^a^		4.65 (4.56, 4.74)^abc^		0.000				
*TG*	2.40 (2.27, 2.52)		1.87 (1.74, 1.99)^a^		1.65 (1.53, 1.78)^ab^		1.42 (1.30, 1.55)^abc^		0.000				
*HDL-C*	1.38 (1.35, 1.40)		1.43 (1.40, 1.45)^a^		1.44 (1.41, 1.47)^a^		1.47 (1.44, 1.50)^a^		0.000				
*LDL-C*	3.19 (3.11, 3.26)		2.99 (2.91, 3.06)^a^		2.82 (2.74, 2.89)^ab^		2.52 (2.44, 2.59)^abc^		0.000					
**Cognition**															
*Visual-spatial and executive*	3.71 (3.59, 3.83)		3.79 (3.68, 3.91)		3.66 (3.55, 3.78)		3.61 (3.49, 3.72)			0.128		
*Naming*	2.86 (2.82, 2.90)		2.90 (2.86, 2.93)		2.91 (2.87, 2.95)		2.86 (2.82, 2.90)			0.210		
*Attention*	5.22 (5.12, 5.33)		5.39 (5.29, 5.48)		5.33 (5.23, 5.43)		5.36 (5.26, 5.46)			0.125		
*Language*	2.03 (1.95, 2.12)		2.09 (2.00, 2.17)		2.02 (1.93, 2.10)		2.00 (1.92, 2.09)			0.534		
*Abstraction*	1.52 (1.46, 1.59)		1.55 (1.49, 1.61)		1.49 (1.43, 1.56)		1.53 (1.47, 1.59)			0.695		
*Memory and delayed recall*	2.66 (2.51, 2.81)		2.98 (2.84, 3.13)^a^		2.73 (2.58, 2.88)^b^		2.74 (2.59, 2.88)			0.011		
*Orientation*	5.72 (5.66, 5.79)		5.81 (5.74, 5.87)		5.86 (5.80, 5.93)^a^		5.83 (5.77, 5.90)^a^			0.020		
*MoCA score*	24.06 (23.66, 24.46)		24.66 (24.27, 25.05)^a^		23.97 (23.57, 24.37)^ab^		24.03 (23.64, 24.42)^b^			0.049		
**Food Items, (g/d)**												
*Fruit*	143.84 (133.40, 154.28)		160.82 (150.68, 170.95)		159.34 (148.86, 169.82)		155.12 (144.94, 165.31)			0.098		
*Vegetable*	296.38 (283.46, 309.30)		306.90 (294.35, 319.44)		306.42 (293.45, 319.40)		302.60 (290.00, 315.21)			0.648		
*Legume*	28.35 (25.75, 30.94)		30.16 (27.64, 32.68)		31.19 (28.58, 33.79)		30.31 (27.78, 32.85)			0.489		
*Cooking oil*	29.06 (27.23, 30.88)		30.94 (29.17, 32.72)		28.70 (26.87, 30.54)		29.81(28.03, 31.59)			0.320		
*Fish*	18.93 (17.34, 20.52)		20.64 (19.10, 22.18)		20.32 (18.72, 21.91)		18.63 (17.08, 20.18)			0.194		
*Whole grain*	31.12 (27.72, 34.52)		35.67 (32.37, 38.97)		37.53 (34.12, 40.95)^a^		42.61 (39.29, 45.92)^ab^			0.000		
*Red meat*	27.84 (2500, 30.68)		29.16 (26.41,31.91)		30.84 (27.99, 33.69)		31.70 (28.93, 34.47)			0.228		
*Poultry*	13.16 (11.77, 14.55)		14.14 (12.80, 15.49)		14.34 (12.95, 15.73)		13.24 (11.88, 14.59)			0.518		
*Nut*	16.61 (14.24, 18.98)		15.67 (13.37, 17.97)		19.49 (17.11, 21.86)		17.07 (14.76, 19.38)			0.138		
*Milk*	125.12 (114.98, 135.26)		118.63 (108.79, 128.47)		135.66 (125.48, 145.84)^b^		137.90 (128.01, 147.79)^b^			0.023		
*Egg*	31.00 (29.20, 32.79)		31.04 (29.30, 32.79)		33.20 (31.40, 35.00)		33.67 (31.92, 35.42)			0.063		

The data were represented as mean (95% CI) or percentage. General Linear Model (GLM) was used for the comparison of serum parameters, cognitive performance and daily dietary intakes. During the comparison of serum parameter, possible confounding factors including gender, age, BMI, smoking habit, alcohol drinking, physical activity, diabetes and hyperlipidemia were adjusted; During the comparison of cognition, confounding factors including gender, age, BMI, smoking habit, physical activity, alcohol drinking, education level and AD family history were adjusted; During comparison of daily dietary intakes, confounding factors including gender, age, BMI, smoking habit, physical activity and alcohol drinking were adjusted. MoCA: Montreal Cognitive Assessment; α-TOH: α-tocopherol; Glu: glucose; TC: total cholesterol; TG: triglyceride; LDL-C: low density lipoprotein cholesterol; HDL-C: high density lipoprotein cholesterol; Q: quartile; a: comparing with Q1 group, *P* < 0.05; b: comparing with Q2 group, *P* < 0.05; c: comparing with Q3 group, *P* < 0.05.

### Serum parameters, cognition and food intake according to α-TOH/retinol ratio

Following the increase of α-TOH/retinol ratio, serum GLU, TC, TG and HDL-C status increased accordingly, while, the LDL-C status exhibited a decreased trend (Table 6). The subjects in Q4 group had the highest GLU, TC, TG and HDL-C status (*P* < 0.01) and the lowest LDL-C status (*P* < 0.05). Cognitive performance decreased according to the increase of α-TOH/retinol ratio. Subjects with Q1 level of α-TOH/retinol ratio had the best cognitive performance in visual-spatial and executive, naming, abstraction (*P* < 0.01), memory and delayed recall domains (*P* < 0.05), and total MoCA score (*P* < 0.05). The best attention and orientation performance were observed in subjects with Q2 or Q3 level of α-TOH/retinol ratio. Following the increase of α-TOH/retinol ratio, daily intake of fruits, whole grains, red meat, nuts, milk and egg increased correspondingly. The highest daily intake of these food items was observed in subjects with Q4 level of α-TOH/retinol ratio. Daily vegetable intake exhibited a decreased trend following the increase of α-TOH/retinol ratio, demonstrating by lower daily vegetable intake in Q3 and Q4 groups.

**Table 6:** Serum parameters, cognition and food intakes according to α-TOH/retinol ratio (n = 1754).

**Parameters, cognition and Food intake**		**α-TOH/retinol ratio**					***P* value**
		**Q1 (n = 431)**	**Q2 (n = 450)**		**Q3 (n = 426)**	**Q4 (n = 447)**	
**Serum parameters (mmol/L)**							
*Glu*	5.64 (5.46, 5.82)			5.96 (5.79, 6.14)	5.91 (5.73, 6.09)^a^	6.21 (6.03, 6.39)^abc^	0.000
*TC*	4.64 (4.55, 4.74)			4.96 (4.87, 5.05)^a^	5.07 (4.98, 5.16)^ab^	5.32 (5.23, 5.41)^abc^	0.000
*TG*	1.48 (1.34, 1.61)			1.80 (1.66, 1.93)^a^	1.87 (1.74, 2.00)^ab^	2.17 (2.04, 2.30)^abc^	0.000
*HDL-C*	1.34 (1.31, 1.37)			1.42 (1.39, 1.45)	1.45 (1.42, 1.47)^ab^	1.51 (1.48, 1.54)^abc^	0.000
*LDL-C*	2.95 (2.87, 3.03)			2.91 (2.83, 2.99)^a^	2.83 (2.75, 2.91)^a^	2.79 (2.71, 2.87)^ab^	0.034
**Cognition**							
*Visual-spatial and executive*	3.88 (3.77, 4.00)			3.82 (3.71, 3.93)^a^	3.64 (3.52, 3.75)^ab^	3.43 (3.32, 3.55)^abc^	0.000
*Naming*	2.94 (2.90, 2.98)			2.86 (2.82, 2.90)^a^	2.87 (2.83, 2.91)^a^	2.86 (2.82, 2.90)^a^	0.000
*Attention*	5.22 (5.12, 5.33)			5.39 (5.29, 5.48)	5.33 (5.23, 5.43)	5.36 (5.26, 5.46)^a^	0.023
*Language*	2.21 (2.12, 2.29)			2.06 (1.98, 2.14)	2.00 (1.92, 2.08)	1.86 (1.77, 1.94)	0.249
*Abstraction*	1.61 (1.54, 1.68)			1.52 (1.45, 1.58)^a^	1.49 (1.43, 1.56)^a^	1.47 (1.40, 1.54)^a^	0.000
*Memory and delayed recall*	3.19 (3.04, 3.33)			2.76 (2.62, 2.90)^a^	2.71 (2.57, 2.85)^a^	2.46 (2.32, 2.60)^ac^	0.021
*Orientation*	5.82 (5.75, 5.89)			5.71 (5.65, 5.78)^a^	5.85 (5.78, 5.91)^b^	5.84 (5.78, 5.91)^b^	0.000
*MoCA score*	25.44 (25.04, 25.84)			24.16 (23.77, 24.55)^a^	23.93 (23.54, 24.32)^a^	23.19 (22.79, 23.58)^abc^	0.012
**Food Item, (g/d)**							
*Fruit*	140.77 (130.28, 151.25)			152.87 (142.58, 163.16)	159.27 (149.08, 169.47)	165.67 (155.40, 175.94)^abc^	0.009
*Vegetable*	332.54 (319.52, 345.55)			297.02 (284.25, 309.79)	288.97 (276.32, 301.61)	293.56 (280.80, 306.30)^abc^	0.000
*Legume*	27.91 (25.29, 30.53)			29.53 (26.96, 32.11)	31.23 (28.68, 33.78)	30.55 (27.98, 33.11)	0.325
*Cooking oil*	31.41 (29.56, 33.25)			28.30 (26.49, 30.12)	29.57 (27.78, 31.37)	29.24 (27.43, 31.05)	0.122
*Fish*	19.57 (17.97, 21.18)			20.15 (18.57, 21.72)	19.66 (18.10, 21.22)	19.04 (17.47, 20.62)	0.816
*Whole grain*	23.77 (20.39, 27.15)			33.98 (30.66, 37.29)	43.18 (39.90, 46.47)^ab^	45.96 (42.65, 49.26)^abc^	0.000
*Red meat*	26.42 (23.55, 29.28)			28.30 (25.49, 31.11)	31.42 (28.63, 34.21)	33.18 (30.37, 35.99)^a^	0.005
*Poultry*	13.67 (12.27, 15.07)			13.30 (11.92, 14.68)	14.48 (13.11, 15.84)	13.26 (11.89, 14.64)	0.575
*Nuts*	12.94 (10.56, 15.32)			14.43 (12.09, 16.76)^a^	19.00 (16.69, 21.31)^a^	21.93 (19.60, 24.26)^a^	0.000
*Milk*	120.38 (110.11, 130.64)			123.00 (112.94, 144.91)	134.94 (124.97, 144.91)	139.20 (129.16, 149.25)^a^	0.029
*Egg*	28.05 (26.25, 29.85)			30.46 (28.69, 32.22)	33.74 (32.00, 35.49)^ab^	36.63 (34.87, 38.39)^abc^	0.000

The data were represented as mean (95% CI) or percentage. General Linear Model (GLM) was used for the comparison of serum parameters, cognitive performance and daily dietary intakes. During the comparison of serum parameter, possible confounding factors including gender, age, BMI, smoking habit, alcohol drinking, physical activity, diabetes and hyperlipidemia were adjusted; during the comparison of cognition, confounding factors including gender, age, BMI, smoking habit, physical activity, alcohol drinking, education level and AD family history were adjusted; During comparison of daily dietary intakes, confounding factors including gender, age, BMI, smoking habit, physical activity and alcohol drinking were adjusted. MoCA: Montreal Cognitive Assessment; α-TOH: α-tocopherol; Glu: glucose; TC: total cholesterol; TG: triglyceride; LDL-C: low density lipoprotein cholesterol; HDL-C: high density lipoprotein cholesterol; Q: quartile; a: comparing with Q1 group, *P* < 0.05; b: comparing with Q2 group, *P* < 0.05; c: comparing with Q3 group, *P* < 0.05.

### Serum parameters, cognition and food intake according to ApoE genotype

Compared to ApoE3 subjects, ApoE2 and E4 carriers demonstrated higher serum TG (*P* < 0.01) and α-TOH concentration (*P* < 0.05). ApoE2 carriers showed to have the highest serum HDL-C (*P* < 0.01) and the lowest LDL-C levels (*P* < 0.01). ApoE4 carriers demonstrated the lowest serum retinol (*P* < 0.05) and lipid-adjusted retinol status (*P* < 0.01), and the highest α-TOH/retinol ratio (*P* < 0.01) as compared to ApoE3 and E2 subjects. In regard to cognition, ApoE4 carriers have lower naming (*P* < 0.05) and orientation abilities (*P* < 0.05) and total MoCA score (*P* < 0.05) than ApoE3 subjects (Table 7).

**Table 7:** Serum parameters, cognition and food intake according to ApoE genotype in the elderly.

**Parameters and cognition**		**ApoE genotype**		***P* value**
	**E3 *(n = 1201)***	**E2 (*n = 249)***	**E4 *(n = 304)***	
**Serum parameters**				
*GLU (mmol/L)*	5.96 (5.85, 6.06)	5.93 (5.69, 6.17)	5.86 (5.65, 6.08)	0.744
*TC (mmol/L)*	4.99 (4.93, 5.05)	4.95 (4.82, 5.07)	5.10 (4.99, 5.22)	0.140
*TG (mmol/L)*	1.73 (1.65, 1.81)	2.08 (1.91, 2.26)^a^	2.01 (1.85, 2.16)^a^	0.000
*HDL-C (mmol/L)*	1.42 (1.41, 1.44)	1.49 (1.45, 1.52)^a^	1.42 (1.39, 1.46)^b^	0.008
*LDL-C (mmol/L)*	2.90 (2.85, 2.95)	2.65 (2.54, 2.75)^a^	2.94 (2.85, 3.04)^b^	0.000
*a-TOH (μmol/L)*	27.00 (26.54, 27.47)	28.23 (27.23, 29.25)^a^	28.02 (27.12, 28.95)^a^	0.025
*γ-TOH (μmol/L)*	4.27 (4.18, 4.39)	4.49 (4.27, 4.73)	4.51 (4.30, 4.70)	0.062
*a-TOH/TG+TC (μmol/mmol)*	4.09 (4.04, 4.16)	4.11 (4.04, 4.27)	4.02 (3.92, 4.13)	0.270
*γ-TOH/TG+TC (μmol/mmol)*	0.65 (0.65, 0.67)	0.67 (0.62, 0.70)	0.65 (0.62, 0.67)	0.708
*Retinol (μmol/L)*	1.95 (1.92, 1.99)	1.92 (1.85, 1.99)	1.85 (1.78, 1.92)^a^	0.020
*Retinol/TG+TC (μmol/mmol)*	0.31 (0.31, 0.31)	0.28 (0.28, 0.31)	0.28 (0.24, 0.28)^a^	0.000
*α-TOH/retinol*	15.42 (15.03, 15.81)	16.23 (15.37, 17.08)	17.04 (16.27, 17.81)^a^	0.001
*γ-TOH/retinol*	2.43 (2.36,2.51)	2.54 (2.37, 2.71)	2.72 (2.56, 2.87)^a^	0.004
**Cognition**				
*Visual-spatial and executive*	3.74 (3.67, 3.81)	3.56 (3.41, 3.71)	3.63 (3.49, 3.76)	0.062
*Naming*	2.90 (2.88, 2.92)	2.87 (2.82, 2.92)	2.83 (2.78, 2.88)^a^	0.032
*Attention*	5.35 (5.29, 5.42)	5.19 (5.06, 5.32)	5.30 (5.18, 5.42)	0.079
*Language*	2.05 (2.00, 2.10)	2.03 (1.92, 2.14)	1.97 (1.87, 2.07)	0.414
*Abstraction*	1.53 (1.49, 1.57)	1.46 (1.37, 1.55)	1.55 (1.47, 1.63)	0.288
*Memory and delayed recall*	2.81 (2.73, 2.90)	2.66 (2.47, 2.86)	2.74 (2.56, 2.91)	0.337
*Orientation*	5.84 (5.80, 5.87)	5.75 (5.67, 5.84)	5.73 (5.65, 5.81)^a^	0.027
*MoCA score*	24.37 (24.13, 24.61)	23.65 (23.11, 24.18)^a^	23.87 (23.39, 24.35)	0.020

The data were represented as mean (95% CI) or percentage. General Linear Model (GLM) was used for the comparison of serum parameters and cognitive performance. During the comparison of serum parameter, possible confounding factors including gender, age, BMI, smoking habit, alcohol drinking, usage of antioxidant supplement, physical activity, diabetes and hyperlipidemia were adjusted; During the comparison of cognition, confounding factors including gender, age, BMI, smoking habit, physical activity, alcohol drinking, education level and AD family history were adjusted. MCI, mild cognitive impairment; TC: total cholesterol; TG: triglyceride; LDL-C: low density lipoprotein cholesterol; HDL-C: high density lipoprotein cholesterol; ApoE: Apolipoprotein E; α-TOH: α-tocopherol; γ-TOH: γ-tocopherol; MoCA: Montreal Cognitive Assessment. *P* < 0.05 was considered to be statistically significant. a: Comparing with ApoE3 subjects, *P* < 0.05; b: comparing with ApoE2 subjects, *P* < 0.05.

### Serum parameters, food intake of normal and MCI subjects according to ApoE genotype

Among normal subjects, the ApoE4 subjects have the highest serum TC and LDL-C levels. The ApoE2 subjects have the lowest serum LDL-C level. ApoE4 subjects exhibited the highest serum α-TOH level and VE/VA ratio (α-TOH/retinol and γ-TOH/retinol), and the lowest lipid-adjusted retinol level. No difference of cognitive performance was found among normal subjects with different ApoE genotypes.

Among MCI subjects, ApoE4 subjects have the highest serum TC, TG, LDL-C, α-TOH levels and VE/VA ratio (α-TOH/retinol and γ-TOH/retinol). The lowest serum HDL-C, retinol and lipid-adjusted retinol levels were also found in ApoE4 subjects. ApoE4 subjects also demonstrated the lowest visual-spatial and executive, naming, attention, language, memory and delayed recall, orientation abilities and total MoCA score. The lowest daily vegetable and fish intakes were also observed in ApoE4 subjects (Table 8).

**Table 8:** Comparison of serum parameters, cognition and food intakes in normal and MCI subjects according to ApoE genotype.

**Parameters and genotype**	**Normal (*n = 1171)***								**MCI (*n = 583)***						***P* value**	
	**ApoE3(n = 812)**	**ApoE2 (n = 151)**				**ApoE4 (n = 208)**		**ApoE3 (n = 389)**		**ApoE2 (n = 98)**			**ApoE4 (n = 96)**			
**Serum parameters**																
*GLU (mmol/L)*	5.89 (5.75, 6.02)		5.89 (5.58, 6.19)		5.72 (5.46, 5.98)		6.08 (5.89, 6.28)		6.02 (5.64, 6.40)			6.22 (5.82, 6.59)			0.167	
*TC (mmol/L)*	4.96 (4.89, 5.03)		4.83 (4.67, 5.00)		5.07 (4.93, 5.21)^b^		5.07 (4.93, 5.21)^b^		5.09 (4.89, 5.30)^b^			5.18 (4.97, 5.39)^b^			0.044	
*TG (mmol/L)*	1.73 (1.64, 1.83)		2.20 (1.97, 2.43)^a^		2.04 (1.84, 2.23)^a^		1.76 (1.61, 1.91)^bc^		1.88 (1.59, 2.16)			1.90 (1.61, 2.19)			0.002	
*HDL-C (mmol/L)*	1.40 (1.38, 1.42)		1.44 (1.40, 1.49)		1.40 (1.36, 1.44)		1.47 (1.44, 1.50)^ac^		1.55 (1.49, 1.61)^abcd^			1.45 (1.39, 1.51)^e^			0.000	
*LDL-C (mmol/L)*	2.96 (2.90, 3.02)			2.62 (2.48, 2.76)^a^	3.00 (2.88, 3.12)^b^			2.82 (2.73, 2.91)^ac^			2.66 (2.49, 2.83)^ac^			2.84 (2.66, 3.01)^c^	0.018	
*a-TOH (μmol/L)*	26.56 (11.20, 11.68)		28.16 (26.86, 29.46)^a^		27.83 (26.72, 28.92)		27.81 (27.00, 28.63)^a^		28.56 (26.93, 30.16)^a^			28.60 (26.98, 30.23)^a^			0.030	
*γ-TOH (μmol/L)*	4.22 (4.10, 4.34)		4.54 (4.25, 4.82)		4.44 (4.20, 4.68)		4.37 (4.20, 4.56)		4.44 (4.08, 4.80)			4.66 (4.30, 5.04)			0.103	
*a-TOH/TG+TC(μmol/mmol)*	4.06 (3.99, 4.11)		4.11 (3.97, 4.27)		3.99 (3.88, 4.13)		4.16 (4.09, 4.27)		4.20 (4.02, 4.39)			4.90 (3.91, 4.27)			0.750	
*γ-TOH/TG+TC(μmol/mmol)*	0.65 (0.62, 0.70)		0.65 (0.62, 0.70)		0.65 (0.60, 0.67)		0.65 (0.63, 0.72)		0.67 (0.63, 0.72)			0.67 (0.63, 0.72)			0.847	
*Retinol (μmol/L)*	2.02 (1.99, 2.06)		1.99 (1.89, 2.09)		1.95 (1.85, 2.02)		1.82 (1.75, 1.85)^ac^		1.82 (1.71, 1.92)^ac^			1.61 (1.50, 1.75)^abcde^			0.000	
*Retinol/TG+TC(μmol/mmol)*	0.31 (0.31, 0.31)		0.31 (0.28, 0.31)		0.28 (0.28, 0.31)^a^		0.28 (0.28, 0.28)^a^		0.28 (0.24, 0.28)^a^			0.24 (0.21, 0.24)^abcde^			0.000	
*α-TOH/retinol*	14.72 (14.24, 15.19)		15.81(14.71, 16.91)		15.97 (15.03, 16.91)^a^		17.06 (16.37, 17.75)^ac^		16.83 (15.47, 18.19)^a^			19.40 (18.03, 20.77)^abcde^			0.000	
*γ-TOH/retinol*	2.32 (2.23, 2.41)		2.49 (2.28, 2.71)		2.50 (2.32, 2.68)		2.69 (2.55, 2.82)^a^		2.63 (2.37, 2.90)a			3.17 (2.84, 3.44)^abcde^			0.000	
**Cognition**																
*Visual-spatial and executive*	4.13 (4.06, 4.20)		3.99 (3.82, 4.15)		4.08 (3.94, 4.22)		2.88 (2.78, 2.99)^ac^		2.89 (2.68, 3.10)^a^			2.65 (2.44, 2.86)^abc^			0.000	
*Naming*	2.94 (2.91, 2.97)		2.96 (2.89, 3.02)		2.96 (2.90, 3.01)		2.80 (2.76, 2.84)^ac^		2.74 (2.66, 2.82)^a^			2.55 (2.47, 2.63)^abcde^			0.000	
*Attention*	5.61 (5.54, 5.68)		5.50 (5.34, 5.66)		5.64 (5.50, 5.77)		4.81 (4.71, 4.90)^ac^		4.73 (4.53, 4.92)^a^			4.57 (4.37, 4.77)^abc^			0.000	
*Language*	2.35 (2.29, 2.40)		2.40 (2.27, 2.51)		2.28 (2.17, 2.38)		1.40 (1.32, 1.47)^ac^		1.46 (1.31, 1.62)^a^			1.31 (1.16, 1.47)^abc^			0.000	
*Abstraction*	1.73 (1.69, 1.77)		1.82 (1.72, 1.92)		1.73 (1.65, 1.81)		1.10 (1.04, 1.16)^ac^		0.89 (0.77, 1.02)^ad^			1.17 (1.04, 1.29)^abce^			0.000	
*Memory and delayed recall*	3.31 (3.22, 3.40)		3.33 (3.12, 3.54)		3.27 (3.09, 3.44)		1.72 (1.59, 1.86)^ac^		1.68 (1.41, 1.94)^a^			1.57 (1.30, 1.83)^abc^			0.000	
*Orientation*	5.94 (5.89, 5.98)		5.96 (5.85, 6.06)		5.90 (5.81, 5.99)		5.61 (5.45, 5.68)^ac^		5.44 (5.31, 5.57)^ad^			5.37 (5.24, 5.50)^abc^			0.000	
*MoCA score*	26.19 (25.98, 26.40)		26.11 (25.62, 26.61)		26.03 (25.61, 26.45)		20.42 (20.11, 20.73)^ac^		19.87 (19.25, 20.49)^a^			19.20 (18.58, 19.82)^abc^			0.000	
**Food item, (g/d)**																
*Fruit*	156.79 (149.11, 164.47)		156.27 (138.55, 173.99)		158.62 (143.31, 173.93)		151.07 (139.71, 162.43)		156.90 (134.59, 179.21)			157.97 (135.56, 180.39)			0.970	
*Vegetable*	312.61 (303.16, 322.06)		311.88 (290.08, 333.67)		308.92 (290.09, 327.75)		293.78 (279.81, 307.76)^a^		281.14 (253.71, 308.58)^a^			272.52 (244.95, 300.09)^abc^			0.018	
*Legume*	29.57 (27.66, 31.47)		32.37 (27.98, 36.76)		28.46 (24.67, 32.25)		30.45 (27.63, 33.26)		28.60 (23.07, 34.13)			31.92 (26.37, 37.47)			0.737	
*Cooking oil*	29.57 (28.24, 30.93)		29.88 (26.78, 32.98)		29.05 (26.37, 31.73)		30.04 (28.02, 31.99)		30.06 (26.15, 33.96)			28.82 (24.89, 32.74)			0.990	
*Fish*	21.00 (19.83, 22.17)		18.44 (15.75, 21,13)		17.50 (15.18, 19.83)^a^		19.45 (17,73, 21.18)		18.29 (14.91, 21.68)			16.10 (12.70, 19.50)^a^			0.016	
*Whole grain*	33.38 (30.89, 35.88)		35.76 (30.01,41.52)		34.90 (29.93, 39.87)		44.63 (40.94, 48.32)^ac^		37.48 (30.23, 44.72)			39.07 (31.79, 46.34)			0.000	
*Red meat*	29.96 (27.87, 32.05)		25.63 (20.80,30.46)		30.80 (26.63, 34.98)		31.65 (28.55, 34.74)		25.25 (19.16, 31.33)			33.57 (27.46, 39.68)			0.156	
*Poultry*	14.60 (13.60, 15.61)		11.65 (9.33,13.97)		13.10 (11.09, 15.10)		13.65 (12.17, 15.14)		11.61 (8.68, 14.53)			11.61 (8.67, 14.55)			0.067	
*Nut*	16.55 (14.80, 18.30)		20.36 (16.33,24.39)		17.04 (13.56, 20.52)		17.13 (14.54, 19.71)		13.61 (8.54, 18.69)			19.85 (14.75, 24.95)			0.324	
*Milk*	127.33 (119.89, 134.77)		129.79 (112.63,146.96)		131.29 (116.46, 146.12)		131.96 (120.95,142.96)		125.51 (103.90,147.13)			139.76 (118.05,161.48)			0.906	
*Egg*	30.76 (29.45, 32.07)		31.02 (28.00,34.03)		33.49 (30.88, 36.09)		34.66 (32.72, 36.59)^a^		36.60 (32.81, 40.40)^a^			30.68 (26.86, 34.49)			0.003	

The data were represented as mean (95% CI) or percentage. General Linear Model (GLM) was used for the comparison of serum parameters, cognitive performance and daily dietary intakes. During the comparison of serum parameter, possible confounding factors including gender, age, BMI, smoking habit, alcohol drinking, usage of antioxidant supplement, physical activity, diabetes and hyperlipidemia were adjusted; During the comparison of cognition, confounding factors including gender, age, BMI, smoking habit, physical activity, alcohol drinking, education level and AD family history were adjusted; During comparison of daily dietary intakes, confounding factors including gender, age, BMI, smoking habit, physical activity and alcohol drinking were adjusted. MCI: mild cognitive impairment; TC: total cholesterol; TG: triglyceride; LDL-C: low density lipoprotein cholesterol; HDL-C: high density lipoprotein cholesterol; α-TOH: α-tocopherol; γ-TOH: γ-tocopherol; ApoE: Apolipoprotein E; MoCA: Montreal Cognitive Assessment. *P* < 0.05 was considered to be statistically significant. a: Comparing with normal-ApoE3 subjects *P* < 0.05; b: comparing with normal-ApoE2 subjects, *P* < 0.05; c: comparing with normal-ApoE4 subjects, *P* < 0.05; d: comparing with MCI-ApoE3 subjects, *P* < 0.05; e: comparing with MCI-ApoE2 subjects, *P* < 0.05.

### Logistic analysis of predictive factors associated with increased risk of MCI

Compared to the subjects with Q1 level of serum α-TOH/retinol ratio, the subjects with Q2, Q3 and Q4 level of serum α-TOH/retinol ratio demonstrated increased risk of MCI (*OR_Q2 to Q1_* = 1.56, *P* = 0.012; *OR_Q3 to Q1_*= 1.87, *P* = 0.001; *OR_Q4 to Q1_* = 2.65, *P* < 0.001). The combined effect of ApoE genotype and serum α-TOH/retinol ratio in affecting the risk of MCI was also observed. ApoE2 carriers with higher serum α-TOH/retinol ratio demonstrated an increased risk of MCI; and for the subjects in Q2 and Q4 groups, the difference was statistically significant (*OR_Q2_* = 2.17, *P* = 0.002; *OR_Q4_* = 1.65, *P* = 0.042). ApoE4 carriers with Q4 level of α-TOH/retinol ratio also demonstrated an increased risk of MCI compared with ApoE3 subjects with Q1 level of α-TOH/retinol ratio (*OR* = 1.89, *P* = 0.004) (Table 9).

**Table 9:** Logistic analysis of ApoE, lipid-adjusted serum α-TOH, retinol status and α-TOH/retinol ratio and the risk of MCI.

**Predictors**	**B**	**SE**	**Wald**	**Adjusted OR**	**95/% CI**	***P* value**
Independent effect of lipid-adjusted α-TOH						
α-TOH/TG+TC Q1 (reference)	-	-	-	1	-	-
α-TOH/TG+TC Q2	-0.083	0.150	0.305	0.920	0.686, 1.236	0.581
α-TOH/TG+TC Q3	0.185	0.150	1.517	1.203	0.897, 1.613	0.218
α-TOH/TG+TC Q4	0.323	0.147	4.841	1.382	1.036, 1.843	0.028
Independent effect of lipid-adjusted retinol						
Retinol/TG+TC Q1 (reference)	-	-	-	1	-	-
Retinol/TG+TC Q2	-0.284	0.144	3.903	0.753	0.568, 0.998	0.048
Retinol/TG+TC Q3	-0.529	0.149	12.517	0.589	0.440, 0.790	0.000
Retinol/TG+TC Q4	-1.193	0.165	56.151	0.303	0.220, 0.419	0.000
Independent effect of α-TOH/retinol ratio						
α-TOH/retinol Q1 (reference)	-	-	-	1	-	-
α-TOH/retinol Q2	0.606	0.162	13.952	1.833	1.334, 2.519	0.000
α-TOH/retinol Q3	0.878	0.163	29.017	2.405	1.748, 3.310	0.000
α-TOH/retinol Q4	1.287	0.164	61.865	3.621	2.628, 4.990	0.000
Synergistic effect of ApoE genotype and lipid-adjusted retinol						
ApoE3 × retinol/TG+TC Q1 (reference) -		-	-	1	-	-
ApoE2 × retinol/TG+TC Q2	0.005	0.298	0.000	1.005	0.560, 1.801	0.988
ApoE2 × retinol/TG+TC Q3	0.488	0.259	3.564	1.629	0.982, 2.705	0.059
ApoE2 × retinol/TG+TC Q4	-0.400	0.314	1.623	0.670	0.362, 1.240	0.203
ApoE4 × retinol/TG+TC Q2	0.157	0.234	0.446	1.170	0.739, 1.852	0.504
ApoE4 × retinol/TG+TC Q3	-0.551	0.305	3.263	0.576	0.317, 1.048	0.071
ApoE4 × retinol/TG+TC Q4	-1.495	0.441	11.484	0.224	0.094, 0.532	0.001
Synergistic effect of ApoE genotype and lipid-adjusted α-TOH						
ApoE3 × α-TOH/TG+TC Q1 (reference) -		-	-	1	-	-
ApoE2 × α-TOH/TG+TC Q2	0.242	0.264	0.835	1.273	0.758, 2.139	0.361
ApoE2 × α-TOH/TG+TC Q3	-0.041	0.302	0.019	0.960	0.531, 1.734	0.891
ApoE2 × α-TOH/TG+TC Q4	0.645	0.251	6.571	1.905	1.164, 3.119	0.010
ApoE4 × α-TOHl/TG+TC Q2	-0.615	0.275	4.997	0.540	0.315, 0.927	0.025
ApoE4 × α-TOH/TG+TC Q3	0.022	0.266	0.007	1.022	0.607, 1.721	0.007
ApoE4 × α-TOH/TG+TC Q4	0.191	0.261	0.531	1.210	0.725, 2.020	0.466
Synergistic effect of ApoE genotype and α-TOH/retinol						
ApoE3 × α-TOH/retinol Q1 (reference) -		-	-	1	-	-
ApoE2 × α-TOH/retinol Q2	0.803	0.259	9.587	2.233	1.343, 3.714	0.002
ApoE2 × α-TOH/retinol Q3	0.534	0.300	3.174	1.706	0.948, 3.071	0.075
ApoE2 × α-TOH/retinol Q4	0.578	0.249	5.389	1.782	1.094, 2.902	0.020
ApoE4 × α-TOH/retinol Q2	-0.106	0.261	0.164	0.685	0.540, 1.499	0.900
ApoE4 × α-TOH/retinol Q3	-0.203	0.281	0.523	0.469	0.471, 1.415	0.816
ApoE4 × α-TOH/retinol Q4	0.694	0.222	9.794	2.002	1.296, 3.093	0.002

Logistic regression models were created to evaluate the independent and synergistic effects of serum α-TOH, retinol, α-TOH/retinol and ApoE genotype on the risk of MCI. Confounding factors such as age, sex, BMI, education, smoking, alcohol drinking, physical activity levels, diabetes and hyperlipidemia were adjusted during analysis. MCI: mild cognitive impairment; α-TOH: α-tocopherol; ApoE: Apolipoprotein E; SE: standard error; OR: odds ratio; CI: confidence interval; Q: quartile.

## DISCUSSION

The relationship between circulating VA status with cognition and dementia remains inconclusive [14,15]. These discrepancies observed between studies may be attributed to the differences in studied populations (community-based population *vs* hospital-based population). In the present study, we found out that a significant positive correlation between circulating retinol status with cognitive performance. Significantly, lower serum retinol content was observed in MCI subjects. Even after adjusting retinol status with lipids, statistical significance was still indicated. The protective effect of increase in circulating retinol status on cognitive function was also elicited by logistical analysis. These outcomes indicate the correlation between circulating retinol status and cognitive function in the elderly. Our data also indicates that subjects with dietary pattern low in vegetables and high in fruits, whole grains, nuts and egg exhibit lower serum retinol status, as well as poor cognitive performance outcomes. Lower daily vegetable intake was also found particularly in MCI subjects. Given that vegetables are rich in VA and other bioactive substances [16], our results highlight the potential role of dietary VA intake in affecting *in vivo* VA nutritional status and consequently, cognitive outcomes.

Progressive neurologic disorders have been found in the patients with VE deficiency [17,18]. Consistent with these findings, our data demonstrate that lower serum α-TOH status correlate with poor cognitive performance. Of note, the best cognitive performance was found in subjects with Q2 or Q3 level of lipid-adjusted α-TOH status instead of in subjects with Q4 level of serum lipid-adjusted α-TOH status. This outcome indicates that higher serum VE status might deteriorate cognition in the elderly, which is further confirmed by a higher serum α-TOH and lipid-adjusted α-TOH status observed in MCI subjects. The relationships between lipids and VE have been comprehensively reported [19]. These results fall in line with the significantly positive correlation between serum α-TOH status and lipid parameters observed in our study (Supplementary materials Table S1). The simultaneous increase in circulating TC, HDL-C and α-TOH status found in MCI subjects further hints the potential role of lipids in the relationship between VE and cognitive function, and may partially explain the inconsistent conclusions derived from different population-based VE supplementation trials [20,21].

In the current study, higher serum α-TOH/retinol ratio was observed in MCI subjects. This higher circulating α-TOH/retinol ratio might attribute to a lipid-rich and vegetable-less diet demonstrating by higher daily fruits, whole grains, red meat, nuts, milk and egg intakes and lower daily vegetable intake in subjects within Q4 quartile of serum α-TOH/retinol ratio. Interactions of VE and VA absorption and tissue accumulation have been reported [22,23], and high dietary levels of vitamin A have been found to depress vitamin E utilization in animals studied [24]. A decline of serum and liver α-TOH was observed in high VA diet fed weaned pigs [25]. These results suggest potential adverse interactions of VA and VE, and an optimal interactive state between VE and VA might be essential to maintain their normal physiological functions *in vivo* [26].

In agreement with other previously published studies [27], increased serum lipids (LDL-C and TG) are observed in ApoE4 subjects. Correspondingly, higher serum α-TOH was also found in ApoE2 and E4 carriers. However, after adjusting α-TOH status with lipid, ApoE genotype difference of VE ceased to establish. These outcomes are consistent with recent results emphasizing that increase in serum TG and LDL-C levels in ApoE2 and ApoE4 subjects might contribute to these genotype-dependent differences observed in serum VE levels [28].

Gómez-Coronado and colleagues found that ApoE polymorphism imposed an independent impact on serum VA levels; and the authors concluded that the potential effect of ApoE2 on VA could not be explained by the increased serum TG levels in ApoE2 subjects [29]. In the current study, we observed an increased serum TG levels and decreased retinol in ApoE2 and E4 subjects. Even after adjusting retinol status with lipids, ApoE genotype difference in retinol status was still significant. These results might be explained by the observed weaker correlation of serum vitamin A with lipids [30]. Poor cognitive performance was found in both ApoE2 and E4 carriers, demonstrated by lower naming ability, orientation ability and total MoCA score. ApoE4-dependent neurological disorder has been extensively reported [31]. The relationship between ApoE2 and neuro-pathologic features of AD has been quite controversial and complex. ApoE2 has suggestively possessed a protective property against cognitive decline [32]. Yet, other investigators have not found any links between ApoE2 and MCI [33]. Therefore, the association between ApoE2 and cognitive function yet remains to be fully clarified.

Direct effect of ApoE on α-TOH dynamics in the brain was strongly suggested by previous studies [34,35]. In the current study, we detected significantly higher serum TC, α-TOH and α-TOH/retinol ratio in ApoE4-MCI subjects. Also, lower daily vegetable, fish and egg intakes and moderate amount of whole grains intake were found in MCI-ApoE4 subjects, which partially indicates the interactive impacts of genetic predisposition (ApoE genotype) and environmental factors (dietary patterns) on lipid profile and cognitive function phenotypes in the elderly. The combined effect of ApoE genotypes and α-TOH/retinol ratio for the risk of developing MCI is also ascertained by the logistic analysis results. In subjects with ApoE2 or E4 genotype, a higher serum α-TOH/retinol ratio predicted an increased risk of developing MCI in the elderly. The outcome of this current study interestingly implicates that the “good” or “bad” roles of ApoE2 or E4 in affecting cognition may depend on both circulating lipids and vitamins (VE and VA) nutritional states.

Conclusively, our findings demonstrate that serum VA and VE states are determined by diet and circulating lipid concentration. The relationship between circulating VE with cognitive performance is also modifiable by lipid status. Lower circulating retinol and higher α-TOH/retinol ratio potentially predict an increased risk for the development of cognitive decline in aging Chinese adults. ApoE2 and E4 carriers with higher circulating α-TOH/retinol states infer poor cognitive performance and an increased risk of developing MCI.

## MATERIALS AND METHODS

### Participants

A total of 1800 Chinese community residents aged 55-80 were randomly recruited from Nanyuan and Wulituo Communities (Beijing, China). Exclusion criteria of the participants were: severe diseases or conditions known to affect cognitive function (e.g., inflammatory diseases, recent history of heart or respiratory failure, chronic liver disease or renal failure, malignant tumors, a recent history of alcohol abuse, history of cerebral apoplexy or cerebral infarction). As per our previously published documents [36], the subjects with AD, Parkinson’s disease (PD), long-term frequency intake of anti-depressants and medication acting on central nervous system, or those unable to finish the cognition tests were also excluded from the study. The Medical Ethics Committee of Capital Medical University (No. 2012SY23) approved the study and written informed consents were obtained from all participants.

### Anthropometric measurements and socio-demographic variables

Anthropometric parameters (height and weight) were measured by registered nurses from the community’s health service center. Body mass indices (BMI) were calculated as weight (kg)/height (m)^2^. Information on demographic characteristics (e.g., age, gender, nationality, and education), lifestyle factors [e.g., living condition (living alone, yes or no), smoking (yes or no), alcohol drinking (yes or no), physical activity (never, 1-3 times/week, 4-5 times/week, everyday), reading habit (yes or no), and housekeeping (yes or no)], AD family history (yes or no), medical history of chronic diseases and the usage of dietary supplements (yes or no) were collected by self-administered questionnaires adopted from our previous studies [37]. Educational level was assessed as the highest level attained and classified into six categories (illiterate, primary school, junior high school, high school, junior college, undergraduate and above).

### Cognitive tests

Global cognitive function was assessed with the Montreal Cognitive Assessment (MoCA) by well-trained medical doctors from the community health service center. According to a previous study conducted in elderly Chinese population, the cut-off points used for MCI diagnosis were as follows: 13/14 for individuals with no formal education, 19/20 for individuals with 1 to 6 years of education, and 24/25 for individuals with 7 or more years of education. The cut-offs above were shown to be sensitive and efficient in the diagnosis of MCI in elderly Chinese population [38].

### Dietary survey

Dietary assessment was carried out according to the description of our previous study [39]. Briefly, the habitual consumption of 11 food groups (fruits, vegetables, whole grains, legume, red meats, poultry, fish, eggs, nuts, cooking oil, milk, comprising 35 items in total) was surveyed by using a validated semi-quantitative food frequency questionnaire (FFQ). The questionnaire was adopted from a questionnaire used for the dietary investigation of Chinese residents [40].

### Blood measurement

#### Measurement of plasma parameters

Fasting venous blood samples were obtained from participants. Blood samples were centrifuged in lithium heparin tubes at 480 g for 10 minutes at 4°C, and then stored at -80°C before further analyses. Plasma glucose (GLU), triglyceride (TG) and total cholesterol (TC) were measured by an ILAB8600 clinical chemistry analyzer (Instrumentation Laboratory Lexington, WI, USA). A commercially available assay from Instrumentation Laboratory (Lexington, WI, USA) was used to determine high density lipoprotein cholesterol (HDL-C). And Low-density lipoprotein cholesterol (LDL-C) was calculated using the Friedewald formula [41]. All samples of each subject were analyzed within a single batch, and the inter-assay coefficients of variation (CV) for all determinations were less than 5%.

#### Measurement of serum retinol and vitamin E

Serum retinol and vitamin E (α-TOH and γ-TOH) concentrations were measured by reverse phase high-performance liquid chromatography (Waters Chromatograph) simultaneously as previously described [42].

#### DNA isolation and genotyping

Peripheral blood samples (6 ml intravenously) were collected in vacuum tubes and stored at -80°C. DNA was extracted from frozen peripheral blood using the Wizare genomic DNA purification kit (Promega, Madison, WI, USA). ApoE genotypes were determined by Polymerase Chain Reaction (PCR) amplification and Restricted Fragment Length Polymorphism (RFLP) analysis according to the method described by Hixson [43]. For ApoE genotype, subjects with the E2/E2 and E2/E3 genotypes were grouped as E2 carrier; subjects with E3/E3 were classified as E3 homozygote; and subjects with E3/E4 or E4/E4 were grouped as E4 carrier.

### Statistical analyses

Data was analyzed with the software SPSS 19.0 (Chicago, IL, USA). Continuous variables were presented as means ± standard deviation (SD) or mean (95% confidence interval, CI). Gender, smoking, alcohol drinking, physical activity, education, AD family history, reading and housekeeping were presented as categorical variables. Participants were classified according to categories of ApoE genotypes and the quartile of serum VE and VA levels. General linear model (GLM) was used to compare the means of the detected parameters and food intake between the groups. The following putative confounding factors were included in the analyses when comparing serum parameters: age, gender, BMI, physical activity, smoking, alcohol drinking, and usage of antioxidant supplement, diabetes and hyperlipidemia. During comparison of daily food intakes, confounding factors including gender, age, BMI, smoking habit, physical activity and alcohol drinking were adjusted. For cognition analysis, factors including gender, age, BMI, education, living condition, AD family history, physical activity, reading and smoking habit, and housekeeping were adjusted. Chi-square test was used for the comparison of binary categorical variables difference among groups. Partial correlation analysis was used to explore the relationship between serum vitamin status with lipids and cognition. Logistic regression model was run to evaluate the risk of cognitive impairment. We adjusted for demographic variables including age, gender, education, smoking, alcohol drinking, diabetes mellitus and hyperlipidemia in the model. Statistical significance was set at *P* < 0.05.

## SUPPLEMENTARY MATERIAL

Table S1
