# Supplementary material for: Diminished circulating retinol and elevated α-TOH/retinol ratio predict an increased risk of cognitive decline in aging Chinese adults, especially in subjects with ApoE2 or ApoE4 genotype
Source: Aging (Albany NY). 2018 Dec 20;10(12):4066–83. doi: 10.18632/aging.101694 (PMC6326676; doi:10.18632/aging.101694)
Supplement: Table S1 [file aging-10-101694-s001.pdf]

Supplementary Table S1. Partial correlation coefficients between serum lipids and VE and retinol status (n = 1754).

Parameters	Retinol	α-TOH	γ-TOH	α-TOH/retinol	γ-TOH/retinol
GLU	-0.034	0.118**	0.099**	0.083**	0.075**
TC	0.020	0.503**	0.275**	0.296**	0.173**
TG	0.109**	0.535**	0.422**	0.240**	0.226**
HDL-C	-0.189**	0.149**	0.019	0.228**	0.117**
LDL-C	0.273**	0.241**	0.158**	-0.036	-0.049*

Partial correlation analysis was used to explore the relationship between serum α-TOH and retinol status with serum GLU and lipids status. Factors including age, gender, BMI, smoking, alcohol and physical activity were adjusted during data analysis. TG: triglyceride; TC: total cholesterol. GLU: glucose; TC: total cholesterol; TG: triglyceride; LDL-C: low density lipoprotein cholesterol; HDL-C: high density lipoprotein cholesterol; α-TOH: α-tocopherol; γ-TOH: γ-tocopherol. *: $P < 0.05$; **: $P < 0.01$.
